# Free Will Emerges From a Multistage Process of Target Assignment and Body-Scheme Recruitment for Free Effector Selection

**DOI:** 10.3389/fpsyg.2019.00388

**Published:** 2019-02-21

**Authors:** Bauke M. de Jong

**Affiliations:** Department of Neurology, University Medical Center Groningen, University of Groningen, Groningen, Netherlands

**Keywords:** embodiment of will, body scheme, free effector selection, parietal cortex, volition

## Abstract

Self-intended action implies an initial stage of assigning an external entity as target of action, with subsequent recruitment of body-scheme information serving the free selection of an appropriate effector system to achieve the action aim. This plurality underscores the concept that neuronal response freedom underlying the generation of such action is not necessarily restricted to a singular cerebral event at its initiation, but that such freedom is embedded in a series of successive processing steps. In this respect, action intention initially concerns the transition of a neutral object into a target of action, while the “will” to act further crystalizes with the recruitment of one’s body scheme. The latter is a prerequisite for effector selection and indeed complements the emerging sense of agency. This temporal order of neuronal events fits a model of fronto-parietal interactions associated with volition. A concise behavioral experiment is additionally described, in which successively displayed balls represent either a recognizable object with distinct shape and color features, or a target of action. Instructions to write down the ball’s characteristics were alternated by the command ”action.” When shifting from a neutral object to an action target, the ball was placed in one of three backgrounds: empty, an outdoor goal or indoor basket. In response to the action command, subjects reported intended actions such as kicking, seizing, throwing and heading, thus implicitly referring to the foot, hand, or head as chosen effector. For the latter the parietal cortex is strongly implicated, not only concerning predefined but also free selection. Although subjects were free to choose what to do with the ball, the environmental cues of the ball strongly influenced their choices. These results illustrate the temporal order in fronto-parietal processing associated with initial target assignment, instantly followed by the embodiment of will, i.e., the recruitment of body-scheme information for possible effector selection. Such multistage neuronal processing underlying free action selection underscores that the onset of brain signals prior to the perceived sense of free will is not a valid argument to reduce free will to an illusion.

## Introduction

### Internal Versus External Action Cues

The generation of self-intended action implies the cerebral organization of goal-directed movements not evoked by an explicit external cue. The prefrontal cortex has been strongly implicated in such function (Frith et al., 1991; Miller and Cohen, 2001; Koechlin and Hyafil, 2007), which is further underscored by symptoms that may arise from prefrontal impairment such as a reduced initiative to undertake internally motivated actions and enhanced responses to external stimuli (Lhermitte, 1983; De Renzi and Barbieri, 1992; Burgess and Shallice, 1996; Godefroy, 2003). Particularly involvement of the medial prefrontal cortex in free action selection has been emphasized (Lau et al., 2006; Mueller et al., 2007; Haggard, 2008; Rushworth, 2008; Zapparoli et al., 2017). This is in line with an influential model dissociating medial and lateral frontal cortex contributions, associated with internally and externally driven motor actions, respectively (Passingham et al., 2010). Regarding the latter, external cues may be seen as an intrinsic part of sensory information about the external world, which is predominantly channeled along indeed the lateral surface of the hemispheres, with strong parietal-frontal interconnections that extend from a dorsal visual pathway (Goodale and Milner, 1992).

On the other hand, self-intended action similarly implies interaction with one’s environment, *also* in the temporal window preceding actual performance. In self-intended action, however, different from externally cued action, an initially neutral constituent of one’s environment is assigned as a target of action, immediately followed by the embodiment of an action plan, i.e., recruitment of a body-scheme for selecting the appropriate motor outflow channels. In the present paper, the distinction of these two aspects of self-intended action (target assignment and free effector selection) is elaborated in order to argue that degrees of response freedom in a neuronal system underlying free action selection is based on a multistage process, of which the recording of brain activity before the perception of a free will to act, need not be in conflict with the concept of consciously intended action.

Without an overt external cue, the initiation of goal directed action within a dynamic environment is strongly influenced by internal reference parameters, enabling the assessment of new environmental circumstances in the context of possible action outcomes. In this, memorized experiences may set the level of reward expected from such possible action (Rolls, 2012). A default mode neuronal system, in which indeed particular medial frontal regions participate, has been postulated to facilitate the shift from an internal steady-state to externally directed action planning (Raichle, 2015). In addition, perceptual analysis of the meaning and/or emotional load of new environmental characteristics, channeled along a (ventral) occipito-temporal visual pathway (Goodale and Milner, 1992) to the orbitofrontal cortex, medial prefrontal cortex and basal ganglia may further reinforce the drive for action (Wager et al., 2005; Carlson et al., 2011; Grabenhorst and Rolls, 2011; Wunderlich et al., 2012; Schultz, 2015, 2016). In other words, perceptual analysis of environmental constituents, framed in the context pre-existing (memorized) information, may define a new target of action. Although it goes beyond the scope of this paper to further elaborate on the neuronal and neurochemical mechanisms implicated in primary sources of internal drives for action, functions and pathways of the monoaminergic transmitter systems might be considered in this respect, given their role in attention, vigilance and arousal (noradrenaline), depression, impulsivity and anxiety (serotonin), reward expectation and motivation (dopamine) (Robert and Benoit, 2008; Westbrook and Braver, 2016). In a psychological context, such an internal driving force has been linked to the concept of conation, i.e., mental energy also required for sustained performance (Reitan and Wolfson, 2000).

The afore described neuronal mechanisms may thus well explain the internal generation of goal directed action, without the necessity of introducing a self-conscious actor as the initiator of voluntary action. On the other hand, the perception of a “free will” to initiate action or refrain from it, as well as the sense of agency, i.e., the feeling of being in control of one’s actions, is an undeniable first-person experience. Although it should be kept in mind that responses of a human subject need to be epistemologically distinguished from responses of neuronal tissue (de Jong, 1989; Bennett et al., 2007), a first challenge to reconcile these different levels of scientific assessment is to find associations between reported perception and distinct cerebral activity underlying self-intended action. The experiment of Libet addressed this issue and revealed that the onset of intention-associated cerebral activity preceded the perception of one’s free will to push a button by means of a finger movement, i.e., the awareness of a non-cued intention to move (Libet et al., 1983; Haggard, 2008). This has been used as an argument that the feeling of voluntary control may be characterized as an illusion (Rolls, 2012): apparently, the sense of agency has no causal relation with the actual initiation of action because it follows such initiation. On the other hand, recent experimental data indicate that neuronal processing underlying free selection involves multiple stages of goal-directed action, both parallel and in sequential order (Cisek and Kalaska, 2010; Cui and Andersen, 2011; Rushworth et al., 2012). This implies that unconscious and conscious decision making may be complementary to and not at odds with each other. Particularly the parietal contribution to decision making provides conceptual arguments for associations between free selection at the level of neuronal processing and the perception of free will and sense of agency at subject level (Sirigu et al., 1999, 2004; Farrer and Frith, 2002; Nahab et al., 2011; Desmurget and Sirigu, 2012).

Regarding the Libet experiment one might oppose that the advocated model of successive stages involved in the initiation of internally generated action, perceived as self-intended, does not directly apply to Libet’s design. Indeed, the latter did not address a natural condition in which participants express spontaneous self-intended actions in interaction with their environment; participants were instructed to make a free choice in time when to push a button. This implies that the task instruction is kept in memory, while the button and a clock are the external objects on which ongoing attention is focussed. In this respect, the Libet experiment is consistent with the postulated multistage model of (free) action intention, in such a way that the initial stage concerns an (unconscious) actualization of the instruction kept in memory (“push the button”) while subsequently, the button becomes an overt target again, with the recruitment of one’s body scheme for (fixed) selection of the finger used to push. This second stage is proposed to be associated with the conscious experience of the self-intended act to move, while a final stage before actual motor execution might address final action consent or veto (internal no-go), mediated by prefrontal interconnections (Chikazoe, 2010; de Jong, 2011; Cieslik et al., 2015).

### Parietal Motor Functions and Sense of Agency

As described in the previous paragraphs, next to the shift of a neutral environmental object into a target of action, a representation of body-scheme is recruited to accommodate free effector selection. At this stage, the parietal cortex is crucially involved. This parietal involvement concerns multiple levels of complexity, ranging from the somatotopic representation of body parts and sensorimotor transformations to the perceived sense of agency. Mapping the spatial relationships between body parts to construct a body scheme (Maravita et al., 2003; Longo et al., 2010; Sereno and Huang, 2014), channeling sensorimotor transformations (Binkofski et al., 1999; de Jong et al., 2001; Freund, 2001; Filimon, 2010; Heed et al., 2015) and the initiation of invariantly instructed motor actions (Andersen, 1995; de Jong et al., 1999; Medendorp et al., 2008; Gallivan et al., 2011) have become well-established parietal functions. More recently, involvement of parietal-premotor circuitry in free selection has highlighted that decision making may already take place at early stages of visuomotor planning, without prefrontal involvement (Platt and Glimcher, 1999; Shadlen and Newsome, 2001; Cisek, 2007; Pesaran et al., 2008; Cisek and Kalaska, 2010). Apparently, the brain employs a strategy of initiating multiple potential motor plans of which one is further elaborated, particularly in conditions of a dynamic environment. This does not only hold for actions that require a selection between distinct environmental targets, but also concerns the variance how to execute a given motor task. The latter is demonstrated by the increased activations in both dorsal premotor and inferior parietal cortices related to free finger selection, contrasted to the invariant condition of fixed finger selection (Beudel and de Jong, 2009).

Beyond its contribution to early-stage decision making, the parietal cortex exhibits unique fields at which direct cortical stimulation may evoke the perceived urge to move a specific body part (Desmurget et al., 2009). The coinciding parietal involvement in both initiating internally generated action and the perception of self-intended action implies that (i) parietal circuitry is characterized by distinct degrees of freedom in neuronal response patterns associated with free motor selection and (ii) this neuronal circuitry can be stimulated in such a way that the perception of self-intended movement arises. Finally, the parietal cortex, in concert with the cerebellum, plays a distinct role in predicting the sensory consequences of goal-directed movement, which is an essential component in motor preparation (McCloskey, 1981; Blakemore and Sirigu, 2003; Poulet and Hedwig, 2007; Beudel et al., 2011; Sokolov et al., 2017). But even more, matching such predicted (feedforward) and actual (feedback) information, dissociated from externally evoked sensation, appears to be a neuronal mechanism associated with the emerging sense of agency, i.e., attributing the effect of a motor action to oneself (Blakemore et al., 1998; Blakemore and Sirigu, 2003; Haggard, 2017). Misalignment of actual and predicted action consequences may result in a reduced sense of agency and even in the illusion of alien action control (Blakemore et al., 2002; Voss et al., 2010). The notion of anticipated sensation may further provide theoretical support for a model of a prospective perception of free will (Metcalfe and Greene, 2007; de Jong, 2011; Chambon et al., 2013).

To summarize, two successive stages were postulated concerning the initiation of internally generated action, and further specified in the preceding paragraphs. The first stage of assigning an environmental “object” to become a target of action, plausibly unconscious, is followed by a stage of organizing the means how to perform such action, i.e., how to give “hands and feet” to an action plan. At this second stage, recruitment of a body-scheme is considered to be a prerequisite for the free selection of an appropriate effector system. The parietal involvement in such effector selection, in the context of the above treated range of parietal functions, provides a strong argument to infer that at this stage, an association can be made between neuronal activity underlying free self-intended action and the perception of a “free will” to initiate such action. While the onset of cerebral activity related to self-intended action thus defines the initial stage, the “second-stage” neuronal activity, which is particularly implicated in free effector selection, and logically associated with the emerging sense of free self-intended action, remains causally involved in the multi-stage process of free selections. In other words, the onset of brain signals prior to the perceived sense of a free will is not a valid argument to reduce free will to an illusion.

## Experimental Illustration

In the following paragraphs, a concise proof-of-principle experiment will be briefly described, illustrating the serial order of segregating (i) the shift from the neutral description of a displayed ball into a target of action, and (ii) the recruitment of body-scheme information for free effector selection. Regarding the latter, the displayed surrounding of the presented ball may implicitly influence the (free) selection of e.g., an arm or a leg to employ performance with the ball.

### Methods of the Experiment

Ten healthy male subjects (one left-hand preference) with a mean age (SD) of 35.3 years (10.7) and ten healthy female subjects aged 35.4 years (13.4), of which two had left-hand preference, participated in the study. Aside from a personal history in sports, no further subject data were filed. According to national regulation in Netherlands, the absence of filing personal details and the fact that the experiment did not inflict a burden on the participants, it was not required to obtain approval from the local Ethics Committee. In line with this strategy to refrain from filing personal data, only oral informed consent was obtained. Participants viewed a computer monitor displaying a power point presentation with a series of 6 pictures, showing a ball that remained at the same position in the center of the successive images. Subjects were informed that various pictures of a ball would be presented, each accompanied by either a question concerning specific features of the ball or the label “action,” written at the bottom of each display. They had to respond by either writing the answer concerning the ball’s characteristics or noting the virtual performance that came to their mind in response to the “action” command. No examples were given, subjects were told that the command for action meant that they had to write down “what they would do.” It is important to understand that, although both conditions in this design include external commands, the “action command” (i) implied the shift from the ball as a neutral object of description into a target of action, and (ii) aimed to test that the obtained answer indeed reflects the recruitment of an effector system, while the actual effector is selected by the participant’s free choice.

**FIGURE 1:**
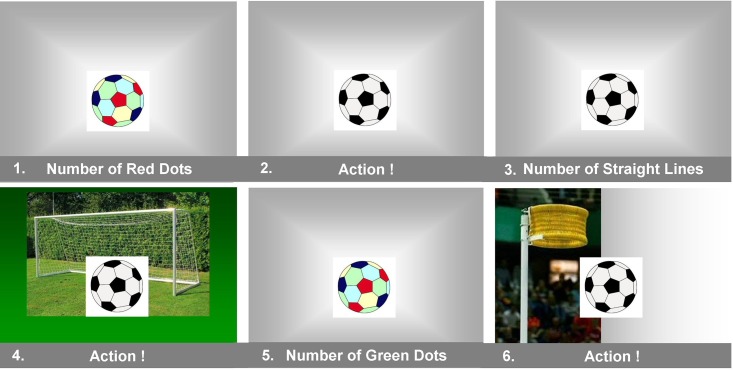
Displays of the ball as neutral object or a target. A series of the 6 displays of the ball is presented in the order 1–6. Subjects were asked to give a specific description of features on the ball’s surface when images 1, 3, and 5 were presented. Images 2, 4, and 6 labeled the ball as a target for virtual (free chosen) action. In the actual PowerPoint presentation used for the experiment, the instructions written at the bottom side of the images 1, 3 and 5 were a bit longer, e.g., image 1 was labeled with “write down the number of red dots you see.”

The series of 6 trials (Figure 1) started with a picture of the ball with its surface marked by adjacent pentagon-shaped dots of various colors, placed on an empty gray background. Subjects had to count the red dots. Next, the ball appeared in black-and-white on the same empty background labeled with the “action” command. The third picture was unchanged, however, subjects had to note the number of straight lines on de ball delineating two adjacent pentagon-shaped dots. Next, the same ball was depicted with an outdoor goal in the background, accompanied by the “action” command at the bottom. The fifth picture was similar to the first, only now subjects had to count the green dots. The final picture with the “action” request framed the black-and-white colored ball with an indoor korfball basket.

### Experimental Results

All 20 subjects provided answers as requested. The descriptions of the ball’s features (questions 1, 3, and 5) were generally correct. The 60 responses to the “action” commands that accompanied displays 2, 4, and 6 are listed in Table 1. Fifty-six responses concerned kicking, seizing, throwing or heading while 4 of 60 responses concerned counting of dots. The background clearly influenced the choice that was made although the selection had remained entirely free. With the basket in the background 19 of 20 subjects responded by the intention to throw the ball. A few subjects commented that, in the empty background, the black-and-white colored ball looked like a football, which was a reason to indicate kicking it away.

**Table 1:** Free self-intended virtual actions.

“Action” Response	Distribution of Actions			Implicit Effector	
Given by particiapants	Background scenery of the bal:				
	No	Outdoor	Indoor
	context	goal	basket	Sum	
# Kicking	15	16	–	Foot	31
# Seizing	3 (2F, 1F*a*)	–	–	Hand	
# Catching	–	1 (M)	–	Hand	
# Throwing	–	1 (F*a*)	19	Hand, sum:	24
# Heading	–	1 (M)	–	Head	1
# Counting black dots	2 (1F, *1Mb)*	1 *(Mb)*	1 *(Mb)*	None	4
Sum	20	20	20		60


*Table 1 lists the answers that were obtained from the 20 subjects in response to the displays of the ball when accompanied by the request for virtual “action.” Before the experiment, subjects were instructed to write down the virtual performance that came to their mind at the appearance of each of the three “action” images. M, male (n = 10); F, female (n = 10); (a) and (b) indicate two distict subjects.*

## Comments on the Experiment

The answers given with regard to the specific features of the ball were based on overt perception of these characteristics, which fits the classical involvement of predominantly the ventral visual pathway (Goodale and Milner, 1992). This involvement may include both bottom-up and top-down information processing (Zeki and Shipp, 1988; Roe et al., 2012). Although the “action” trails were dominated by virtual performance, more covert perception of the ball’s background clearly influenced the chosen virtual action. In the initial phase of action initiation, with the perceptual shift from the ball as a subject of visual assessment into a target of action, its visual surrounding thus unconsciously added further qualification of the target. In the next stage, to achieve the intended action, a hand, foot, or the head was implicitly chosen for virtually performing kicking, seizing, throwing, or heading. This embodiment of an action plan required the recruitment of one’s body scheme. In other words, action aimed at the ball, an external object, was now linked to a specific body part, which implies a link to oneself. It is therefore at this stage, with the above argued involvement of particularly parietal functions, that free effector selection may be logically associated with the emerging perception of a free will of making such selection.

The covert influence of the ball’s visual environment on free effector selection points at interactions between the ventral and dorsal visual pathways. Such interactions may be based on direct temporal–parietal interconnection or mediated along temporal–prefrontal pathways toward parietal circuitry (Goodale, 2011; van Polanen and Davare, 2015; Milner, 2017). The presented experiment with a single object (the ball) placed in various conditions, differed from the more natural circumstance with multiple environmental objects, of which one is assigned as a target of action. Although the experimental and natural circumstances both include the shift from a neutral object into a target of action, there was no selection between objects in the experiment. Unconscious object selection in natural circumstances may be stronger embedded in a ventral visual pathway fuelling top-down prefrontal-parietal interactions (Grabenhorst and Rolls, 2011; Wunderlich et al., 2012), compared to the more variable temporal-parietal interaction assumed in this experiment (van Polanen and Davare, 2015). Not only the ball’s background influenced the choice for action. Without background features, the surface pattern of the ball may have suggested a football, explaining the preference for kicking. In response to e.g., a displayed bowling ball, kicking would have been an unlikely choice.

## Conclusion

At the initiation of free self-intended action, a succession of multiple neuronal processing steps may be discerned. An initial stage of unconsciously assigning a neutral object to become a target of action is followed by a second stage of body-scheme recruitment, enabling the selection of an effector to achieve the intended action. While initially, intention is aimed at the external target, the embodiment of an action plan represents the subsequent linkage between the intended action and a specific body part, thus linking the action to oneself. At this stage, when particularly parietal functions are involved, free effector selection may be logically associated with the emerging sense of agency and perception of a free will in action control. This postulated multistage neuronal processing underlying free action selection underscores that the onset of brain signals prior to the perceived sense of free will is not a valid argument to reduce free will to an illusion. A multistage model, as presented in this paper, may further lay ground for novel experimental designs to explore various steps in the association between internally generated action and the perception of such self-intended action, aimed at providing a more accurate, and undisputed definition of free will.

## Author Contributions

The author confirms being the sole contributor of this work and has approved it for publication.

## Conflict of Interest Statement

The author declares that the research was conducted in the absence of any commercial or financial relationships that could be construed as a potential conflict of interest.
